# Long-Distance Electron Transfer by *G. sulfurreducens* Biofilms Results in Accumulation of Reduced *c*-Type Cytochromes

**DOI:** 10.1002/cssc.201100734

**Published:** 2012-05-10

**Authors:** Ying Liu, Daniel R Bond

**Affiliations:** a BioTechnology Institute and Department of Microbiology, University of Minnesota140 Gortner Laboratory, 1479 Gortner Ave St. Paul MN 55108 (USA)

**Keywords:** bacteria, cytochrome, fuel cells, redox hydrogel, spectroelectrochemistry

The *Geobacteraceae* group of bacteria possesses a natural ability to link cytoplasmic metabolism with redox chemistry at their external surface. Although this capability likely evolved to take advantage of environmental metal oxides as electron acceptors,^1^ it fortuitously allows collection of electrical current from these bacteria.^2^ To transfer electrons from the cytoplasm to acceptors beyond the external membrane, successful metal-reducing bacteria solve multiple biophysical challenges. Electrons produced by oxidative intracellular reactions are inserted into the cytoplasmic membrane and then transferred over 100 Å across the cell wall and outer membrane, where redox proteins must then interact with an unpredictable array of metal oxides. In addition to relaying electrons between daughter cells after cell division that grow as multicellular communities or access highly irregular surfaces, there is a need for longer-distance electrical connections that extend many microns in scale.^3^–^6^

Conceptual models of electron transfer between cells and through *Geobacter* biofilms vary widely. Some data suggests that electrons travel via protein fibers with metallic-like conductivity,^7^ whereas other evidence supports a model involving exchange of electrons between cytochromes organized along protein and polysaccharide scaffolds.^8^, ^9^ Each of these models are built upon the phenomenological observation that electrons travel tens of microns through a biofilm, but both lack data on the status of proteins within the biofilm, which could place constraints on key events.

The use of spectroscopic methods during potentiometric analysis has provided a new tool to directly measure the redox status of multiple cofactors in electrode-reducing bacteria.^10^–^13^ Two recent studies specifically addressed construction of spectroelectrochemical reactors able to monitor reduction states of *c-*type cytochromes in *Geobacter sulfurreducens* (*G. sulfurreducens*) biofilms while maintaining physiological conditions, whereas another focused on the cell–electrode interface. In these cases, fully grown biofilms were the primary target of these noninvasive measurements. The effect of biofilm thickness on the kinetics of electron transfer to *c*-type cytochromes has not been compared or addressed in light of recent electron-transfer models.

The purpose of these experiments was to continuously monitor the cytochrome redox status during active respiration in the presence of electron donors, as well as in biofilms depleted of substrates. These observations detect an accumulation of electrons, which occurs within *G. sulfurreducens c*-type cytochromes, but only after multiple cell layers have formed on the electrode surface. By removing electron donors and altering electrode potentials at progressively more rapid rates, the *c*-type cytochromes are shown to rapidly equilibrate with electrodes only when biofilms are under a few cell layers thick. Oxidation of *c*-type cytochromes lags significantly behind changes in electrode potential when biofilms are on the order of 10–20 μm thick, suggesting that electron transfer to and from cytochromes within the biofilm represents a rate-limiting step to electron transfer. The inability of stronger driving forces to accelerate electron transfer shows that these slower reactions are not directly connected to the electrode.

In a previous work, the relationship between spectral characteristics of *G. sulfurreducens* and imposed potential was determined for fully grown biofilms (>20 μm).^12^, ^13^ For this study, biofilms were investigated at various stages of growth, in both, the presence and absence of the electron donor acetate. In all experiments, cells were introduced into the stirred, anaerobic spectroelectrochemical chamber at 30 °C with 30 mm acetate, allowed to attach to the indium tin oxide (ITO) electrode poised at +0.24 V for up to 12 h; then, planktonic cells were removed, and the biofilm was allowed to grow in the presence of 30 mm acetate.

As *G. sulfurreducens* current densities increased from 50 to nearly 500 μA cm^−2^, planktonic cell density did not increase above an optical density (at 600 nm; OD^600^) of 0.05, while the absorbance of the biofilm increased by over 1 OD unit. In addition, removal of the electrode and all planktonic cells revealed that less than 1 % of this absorbance was due to growth of cells on walls of the cuvette, as had been previously observed.^13^ Thus, virtually all of the spectral data collected during these experiments was from electrode-attached cells.

During biofilm growth with an electrode potential of +0.24 V, the electrode was periodically poised at −0.35 V, a potential known to bring all *c-*type cytochromes to 100 % reduction.^12^, ^13^ Comparison of spectral scans revealed the surprising result that, after the biofilm progressed beyond ≍200 μA cm^−2^, a percentage of biofilm *c-*type cytochromes were in the reduced state even in the presence of an oxidizing electrode (Figure 1). The magnitude of this electron accumulation within the respiring biofilm increased with current density. Increasing the electrode potential to +0.5 V could not relieve or in any way alter this partially reduced state. The buildup of electrons in the biofilm was observed for all replicates (*n*=4) and was also similar to data recently published for late-stage biofilms by Jain et al.^12^

**Figure 1:**
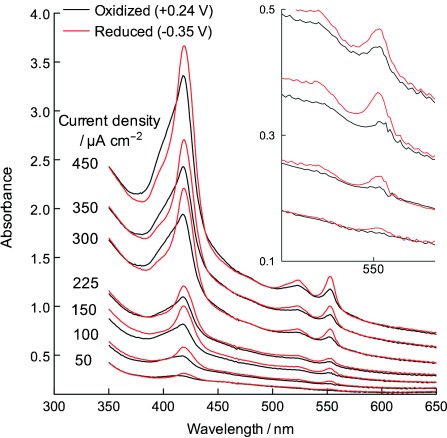
Absorbance spectra of *G. sulfurreducens* biofilms while growing at oxidizing (+0.24 V) potentials (black traces). After collecting each scan, electrodes were poised at −0.35 V for 10 min to obtain data from the fully reduced state (red trace). Application of potentials as high as +0.5 V did not further oxidize cytochromes during growth. Inset expands data from 50–225 μA cm^−2^. All potentials are versus standard hydrogen electrode (SHE).

Although planktonic cells, and cells on the cuvette wall, had been eliminated as causes of this reduced cytochrome signal, it remained possible that cells on or in the biofilm could be ‘disconnected’ from the electrode, dead, or otherwise unable to use the electrode as acceptor. To test this hypothesis, a series of spectral scans were collected from a mature biofilm producing 450 μA cm^−2^ (Figure 2 A) in the presence of acetate; then, acetate was removed from the medium surrounding the electrode, and the experiment was conducted again (Figure 2 B).

**Figure 2:**
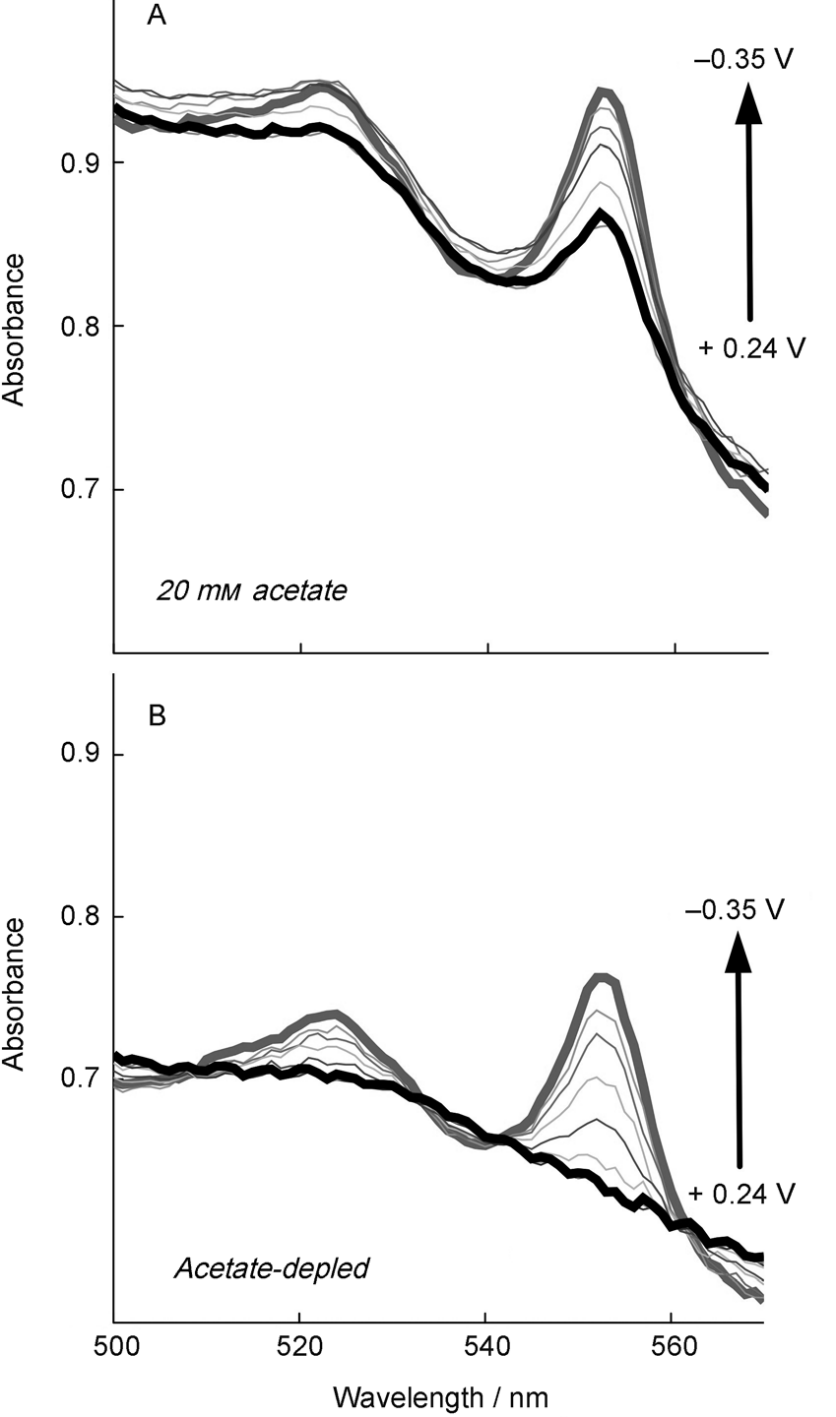
Absorbance data from *G. sulfurreducens* producing 450 μA cm^−2^, a stage at which the biofilm extends beyond 20 μm and growth slows significantly. A) Data are at potential intervals of 100 mV and in the presence of acetate. B) Data are from the same biofilm, exposed to the same potentials, in the absence of acetate. These experiments show that all cells were able to discharge electrons to the electrode.

When acetate was removed, application of an oxidizing potential (+0.24 V) always produced the signature of completely oxidized *c-*type cytochromes, that is, the absence of a peak at 522 and 552 nm and full shifting of the Soret band. Identical results were obtained if acetate was allowed to naturally deplete from the biofilm over a 12 h period, indicating that this was not due to washing of cells from the biofilm or reactor. This data proved that the electron accumulation during growth of the biofilm was occurring in cells that were electrically connected to the electrode.

A second source of evidence that the biofilm on the electrode was uniform and not consisting of dead cells or interfering substances was the relationship between total cytochromes and biomass. When the height of peaks obtained during full reduction (at 552 nm) was compared to the increase in OD^600^, it was possible to monitor the cytochrome/biomass ratio. This value remained constant throughout the growth phase (from 50 to 500 μA cm^−2^).

The increase in reduced cytochromes was also dependent on the concentration of acetate, and thus the rate of respiration. When acetate was added stepwise to acetate-depleted biofilms of *G. sulfurreducens,* the current density also increased stepwise until acetate concentrations exceeded 1 mm.^14^ With each acetate addition, absorbance of characteristic peaks indicative of reduced *c-*type cytochromes increased, showing that this effect was reversible (Figure 3). As this effect did not require high levels of acetate to be evident, it was not merely an issue of electron ‘overflow’ at abnormally high rates of electron transfer, but occurred at all rates of respiration.

**Figure 3:**
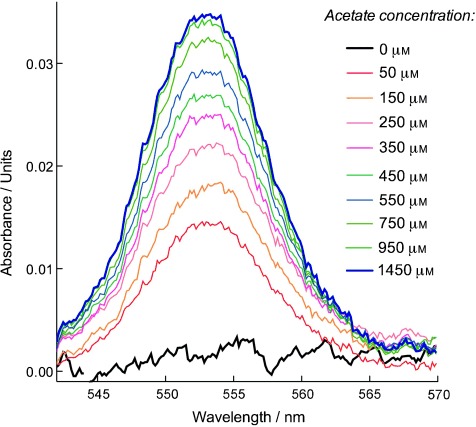
Absorbance data of a *G. sulfurreducens* biofilm depleted of electron donors at a current density of 450 μA cm^−2^. While poising the electrode at +0.24 V, acetate was added stepwise at 15 min intervals. Even when the respiration rate was less than 10 % of maximum (<100 μm acetate), accumulation of electrons in cytochromes was still detected.

The fact that *c-*type cytochromes produce such a well-defined spectral pattern in the 552 nm range, with a flat baseline spanning two isosbestic points under completely oxidized conditions, makes it possible to estimate the degree of cytochrome reduction at any given point if a measurement under fully reducing conditions is available. For example, in Figure 2 A, the absorbance of cytochromes at fully reduced potentials can be measured directly. Then, using an interpolated baseline between isosbestic points, it can be estimated that nearly 50 % of the cytochrome pool remains reduced even when the electrode is at +0.24 V. Using this approach, it was possible to rapidly sample three wavelengths to measure the degree of cytochrome reduction during continuous techniques such as cyclic voltammetry.

Data showing the relationship between cytochrome redox status and potential, for respiring *G. sulfurreducens* biofilms at different stages of growth, is presented in Figure 4. Thin biofilms represent an early stage of growth (<100 μA cm^−2^), at which a biofilm 1–2 cells deep covers the entire electrode. Thick biofilms were obtained at later stages of growth (>450 μA cm^−2^), at which point cell layers extend at least 20 μm from the electrode. This relationship between current density and biofilm thickness has been reported in multiple electrochemical experiments,^14^–^17^ and coverage of electrodes was verified after each experiment reported here via confocal microscopy (data not shown). Figure 4 A shows current production by representative biofilms as the electrode potential is slowly (0.5 mV s^−1^) swept back and forth between low and high values, producing the characteristic sigmoidal catalytic wave differing only in magnitude, which reflects the biomass difference between the two electrodes.^15^–^19^

**Figure 4:**
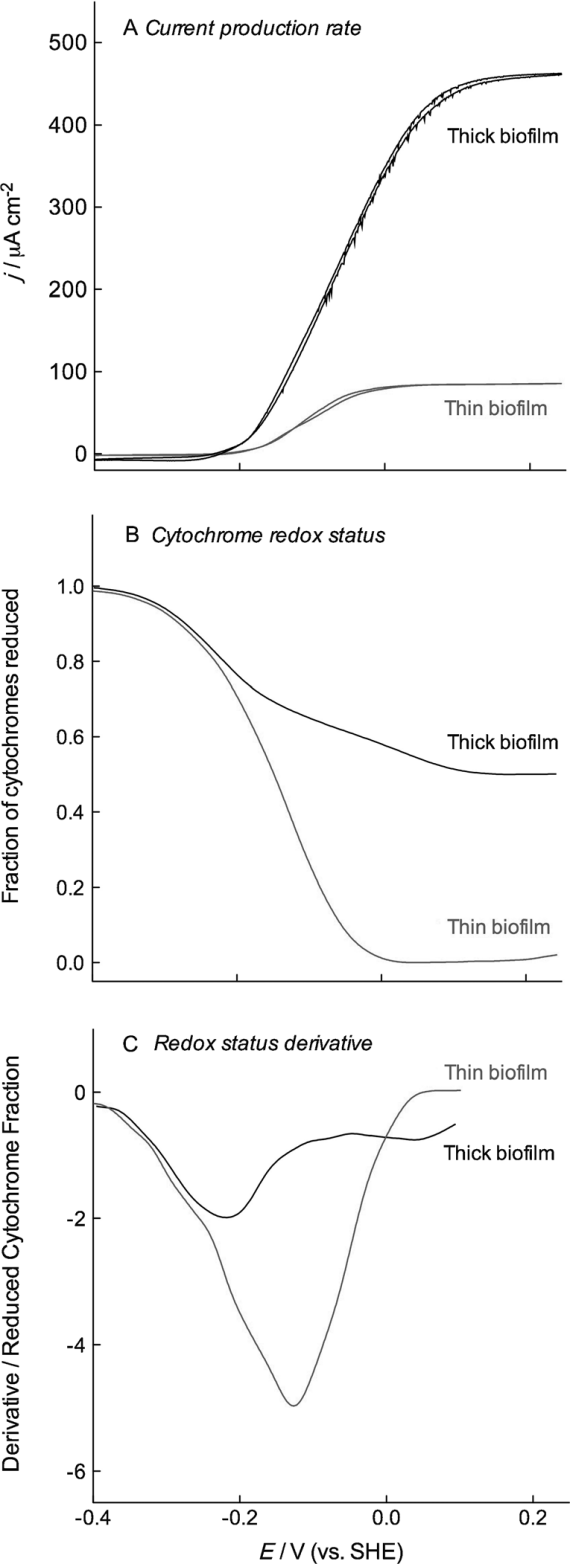
Continuous monitoring of *c*-type cytochrome absorbance during CV shows potentials at which thick biofilms (over 20 μm thick) experience electron accumulation. CVs (0.5 mV s^−1^) of biofilms in (A) are compared to cytochrome redox status in (B). The derivative (C) highlights similarities at lower potentials and the failure of thicker biofilms to respond to applied potentials in the −0.1 V range.

During these potential sweeps, absorbance was continuously monitored at 552 nm, as well as at the isosbestic points of 542 and 562 nm. These experiments verified the observations first shown in Figures 1 and 2; thin biofilms clearly equilibrated with the electrode at all potentials (oxidizing all cytochromes), whereas thicker biofilms failed to completely oxidize regardless of electrode potential. By continuously monitoring changes during cyclic voltammetry, a potential range could be identified where electrons appeared to accumulate.

Both thick and thin biofilms demonstrated similar behavior at lower potentials, each oxidizing approximately 25 % when the potential was raised above −0.2 V. However, while thin biofilms continued to oxidize in the −0.15 to −0.05 V range, reaching 100 % oxidation by 0 V, thicker biofilms only partially oxidized. This data suggested that the backlog of electrons was not occurring in the full suite of cytochromes expressed by *G. sulfurreducens*, but only in a subset with potentials centered around −100 mV. These experiments, performed with actively respiring cells producing electrons throughout the biofilm, reveal a bottleneck that limits electron transfer to the electrode, with some fraction of the cytochrome pool upstream of this bottleneck.

To probe the kinetics of this further, biofilms grown to either thin (100 μA cm^−2^), or thick (450 μA cm^−2^) stages of growth, were washed free of acetate and subjected to cyclic voltammetry at progressively faster scan rates, while again continuously monitoring the cytochrome redox status. In this way, electrons were introduced into the biofilm from a single location (the base), and cytochrome redox status was used to indicate if all cytochromes were able to reach the potential of the electrode on increasingly faster timescales.

For thin biofilms, the oxidation state of *c-*type cytochromes remained a function of imposed potential, even when scan rates were increased (Figure 5 A and B). At scan rates of 20 mV s^−1^, no significant hysteresis or deviation was detected. By plotting the derivative of the redox profile, only a slight flattening of the response was evident, but both forward and reverse scans remained centered on the same midpoint potential. These observations were consistent with all steps in electron transfer being rapid enough to bring all cytochromes to the same potential as the electrode, even when the potential was changed 40-fold faster.

**Figure 5:**
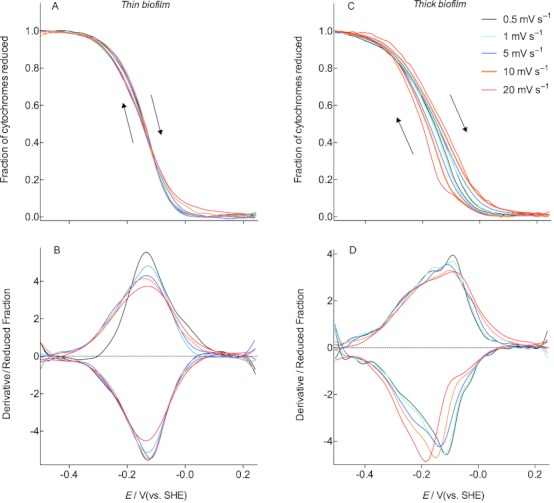
Voltabsorptometry profiles for *G. sulfurreducens* biofilms less than 5 μm thick which were depleted of acetate. Data in (A) shows how cytochrome redox status remains similar as scan rate is increased, irrespective of direction, and the derivative in (B) reveals the slight broadening of the response without peak shifting. C, D) Voltabsorptometry profiles for *G. sulfurreducens* biofilms 20 μm thick, which were depleted of acetate. Beyond scan rates of 1 mV s^−1^, *c-*type cytochrome absorbance lagged behind changes in electrode potential, producing the hysteresis in the higher potential window in (C), and peak shifting of the derivative in (D).

In contrast, the redox status of cytochromes in thicker biofilms lagged significantly behind changes in electrode potential, even above scan rates of 1 mV s^−1^ (Figure 5 C and D). Interestingly, as had been observed in Figure 4, the lag was not seen throughout the entire potential window, but largely within the potential range spanning −0.15 to −0.05 V. This lag in cytochrome reduction could be easily visualized in the derivative of the redox profile, where the midpoint potentials of cytochrome oxidation separated with scan rate, analogous to how voltammetry peaks deviate with scan rate.

Although such peak movement has been previously observed during CV of *Geobacter* biofilms,^15^–^19^ the source of these electrons was not clear. The absorbance profiles of *c-*type cytochromes within the biofilm demonstrated that slow kinetics of cytochrome oxidation and reduction were responsible for these electrochemical observations. These results reveal that a rate-limiting step to electron transfer in an approximately 20 μm thick biofilm exists that prevents cytochromes from rapidly reaching the same potential as the electrode. As shown by experiments in the presence of acetate, this rate-limiting step cannot be overcome simply by applying a stronger potential.

As the lag was primarily seen in a higher potential range during CV, the multiple peaks observed in *G. sulfurreducens* biofilms may reflect different cytochromes with different kinetics. For example, the ‘slow’ high-potential peak shifts as scan rate is raised and merges with other peaks that do not shift as fast as they are derived from ‘faster’ reactions. Such independent peak movement may confound electrochemical measurements aimed at tracking peak areas or location.

During the initial phases of colonization, when the electrode surface is available, transfer of electrons from *G. sulfurreducens* to an electrode shows no evidence of limiting respiration. The first cells to attach are able to double as fast as cells respiring soluble Fe^III^, and electron transfer rates per unit protein remain constant as new cells are added;^15^–^19^ the work presented here shows that all cytochromes can be oxidized by the electrode. Such data reveals that the series of steps involving transfer of electrons across membranes, coupled to all interfacial reactions linking membranes to external electrodes, are not rate limiting for individual *G. sulfurreducens* cells utilizing graphite electrodes.

According to both, confocal microscopy measurements and calculations of cell packing, smooth electrodes are completely colonized by a monolayer of *G. sulfurreducens* cells at current densities of approximately 100 μA cm^−2^.^14^–^17^ Yet, even as cells grow beyond the point where contact is no longer possible, these new cell layers are also able to actively respire, leading to the conclusion that subsequent biofilm layers possess some conductivity.^6^, ^15^ This conductivity is confirmed by the behavior of biofilms grown on thin gold strips,^14^ across electrode gaps,^20^ and on interdigitated electrodes.^8^ The observations presented in this work show that some aspect of this conductivity becomes limiting as the film extends outward, which manifests as an accumulation of electrons in *c-*type cytochromes within the biofilm (Figure 1).

The accumulation of electrons within cytochromes under steady-state conditions, when cells are respiring acetate and an oxidizing electrode is available, raises the question of where these cytochromes are located. If proteins were at the electrode interface, or if they were connected to the electrode via a mechanism able to immediately transmit electrode potential to the cytochromes, simply increasing the potential would alleviate this problem. However, no amount of additional driving force is able to eliminate this bottleneck, showing that these proteins are distant from the electrode and in electrical contact via a mechanism isolated from the electrode potential.

Multiple hypotheses have described models of electron transfer between cells in *G. sulfurreducens* biofilms. These range from metallic networks,^7^ which increase electron transfer rate as a linear function of imposed potential, to redox polymer-like assemblies, which require concentration gradients to drive a diffusional electron flux between redox centers.^8^, ^9^ Electrochemical experiments with living *G. sulfurreducens* biofilms have repeatedly observed evidence for diffusional behavior via scan rate analysis,^13^, ^14^, ^18^, ^19^, ^21^ electrochemical impedance spectroscopy,^17^ and source–drain electrode experiments.^8^ The spectroelectrochemical data collected under both growth conditions (Figures 1–4) and in acetate-depleted cells (Figure 5) are consistent with observations noting diffusional limitations.

It is important to note that these experiments detect the existence of rate-limiting reactions within the biofilm that cannot be accelerated by voltage, while the presence or absence of internal conductive regions linking such reactions cannot be addressed per se. For example, if electrons travel short distances in clusters of highly conductive pathways, but have to exchange between these regions via slower cytochrome–cytochrome interactions, cytochrome oxidation and reduction would appear as a rate-limiting step, and additional potential could not accelerate these collisions. Regardless of the model, this spectral data reveals that the majority of *c-*type cytochromes within the *G. sulfurreducens* biofilm are discharged via physical events that do not respond to changes in potential as if they were directly wired to the electrode.

Electron transfer between redox centers is strongly affected by the physical mobility, reactivity, and location of redox-active sites, as well as the mobility of counterions.^22^–^24^ The total sum of all physical interactions can be expressed as an ‘apparent diffusion coefficient′, and the approach of Rusling and Forster^24^ provides a useful tool to describe films similar to *G. sulfurreducens.*

The dimensionless parameter *D*_CT_*λ*/*d*^2^ describes a simple ratio between the distance electrons can travel over time and the thickness of a film. *D*_CT_ is the apparent electron diffusion coefficient (in cm^2^ s^−1^), *d* is the film thickness (in cm), and *λ* is the experimental time scale, (in seconds, *λ*=*RT*/*Fν* in CV; *R*, *T*, *F*, and *ν* correspond to the gas constant, temperature, Faraday constant, and frequency, respectively). If this ratio is significantly greater than 1, electrons can migrate fast enough to reach the base of the biofilm before the electrode potential is changed. Under conditions where *D*_CT_*λ*/*d*^2^ is less than 1, the potential of the electrode is changed faster than electrons can travel through the film, a gradient will exist, and some regions will not be at the same potential as the electrode. The electrons eventually arrive at the electrode a short time later, causing the apparent midpoint potential of oxidation or reduction to shift.

As *Geobacter* films of >20 μm show characteristic diffusional behavior at scan rates above 1 mV s^−1^,^8^, ^13^, ^14^, ^18^, ^19^, ^21^ the electron diffusion coefficient has to be on the order of 1–10×10^−8^ cm^2^ s^−1^. This rate is considerably slower than rates of counterion diffusion, suggesting that it reflects protein–protein interactions and that it is not simply an artifact of working in an electrolyte system. Such rates of apparent electron migration are easily achieved in redox polymer systems without addition of other conductive agents.^25^–^28^

When the biofilm is thinner, on the order of 5 μm or less, this same rate of electron migration is fast enough to explain the observation that cells are in equilibrium with the electrode. This highlights the exponential effect that distance has when diffusion is involved. A film a few cells thick can have diffusional events controlling electron transfer, but will not experience any limitations. A doubling in film thickness, however, can suddenly make the distance insurmountable. This steep thickness dependence agrees with our observations in Figures 4 and 5 and may help to explain why *G. sulfurreducens* biofilm growth suddenly slows at this point.

Finally, it is notable that the accumulation of electrons within *G. sulfurreducens* biofilms is not merely an artifact of rapid electron-transfer rates, which may be expected to cause ‘overflow’ into the cytochrome pool.^29^ Electrons accumulated even at very slow rates of electron transfer, that is, when only micromolar concentrations of acetate were provided (Figure 3). This important distinction is consistent with a gradient being a requirement for long-range electron transfer.^24^, ^30^ In diffusion, a gradient is the only way to achieve directional flux.^31^

In summary, by monitoring the redox status of *G. sulfurreducens c-*type cytochromes, a buildup of electrons within cytochromes was observed as biofilm growth progressed beyond a few cell thicknesses. This buildup was not uniform across all cytochromes, but was particularly focused in the potential range of −0.1 to −0.05 V versus SHE, the upper potential window of *c-*type cytochromes known to be exposed to the outer surface.^32^–^37^ As application of stronger potentials could not relieve this issue, these cytochromes did not appear to be linked to the electrode via direct metallic-like connections.

When the redox status of cytochromes was monitored during increasingly rapid changes in electrode potential using non-respiring cells, reduction of cytochromes also lagged behind potential changes when films were multiple cell layers thick, as would be expected if diffusional kinetics controlled cytochrome reduction. The behavior seen in these experiments agrees with recent modeling by Strycharz,^31^ who concluded that the electrochemical response of *Geobacter* biofilms was consistent with a multi-step diffusional process limited by transfer of electrons out of the bacterium, which would result in a gradient of reduced cytochromes extending away from the electrode surface. Lacking difficult-to-quantify variables such as the concentration and location of key proteins, their physical mobility, the contribution of microscopic percolation networks,^30^ or the possibility of localized electron tunneling,^7^ all models of electron transfer through the *G. sulfurreducens* supramolecular network remain speculative. However, by identifying the causes of steps rate-limiting to electron transfer within this network, improvements to biofilm conductivity may not require such exhaustive knowledge.

## Experimental Section

**Bacterial strain, culture media, and biofilm growth:** *G. sulfurreducens* strain PCA (ATCC51573) was subcultured in our lab at 30 °C using a vitamin-free anaerobic medium as previously described. Acetate was provided as an electron donor at 30 mm. All media were adjusted to pH 6.8 prior to the addition of NaHCO_3_ (2 g L^−1^) and were flushed with oxygen-free N_2_/CO_2_ (80:20 v/v) prior to sealing with butyl rubber stoppers. All experiments were initiated by inoculating with 40 % (v/v) of cells within 3 h of reaching maximum optical density (OD^600^>0.6) in the medium described, with fumarate (40 mm) as the electron acceptor.

**Electrochemistry and UV/Vis spectroscopy:** Chronoamperometry (CA) was performed by using a 16-channel potentiostat, and cyclic voltammetry (CV) was conducted by using a Gamry PCI4 Femtostat. Low-resistance indium tin oxide (ITO)-coated glass was obtained from Bayview Optics (Maine) with a working area of 2 cm^2^, which was cleaned in acetone and deionized water, respectively. The ITO electrodes were used as working electrodes. Pt wires and saturated calomel reference electrodes (SCE, 0.244 V vs. SHE) were used as counter electrode and reference electrode, respectively. All reported potentials are versus SHE. The headspace of the cuvette was continuously flushed with oxygen-free N_2_/CO_2_ (80:20 v/v), and all experiments were conducted at 30 °C. A Cary 50 Bio UV/Vis spectrophotometer (Varian) was used to measure the UV/Vis spectrum by using a quartz cuvette of 10 mm path length (Starna Cells, Atascadero, CA, USA) fused to a top that could be sealed with a butyl stopper to exclude oxygen. A magnetic stir bar and stirring cuvette holder ensured constant mixing.

**Confocal microscopy and scanning electron microscopy (SEM) analysis:** After each experiment, biofilms on electrodes were sacrificed, stained with the LIVE/DEAD Baclight bacterial viability kit (Invitrogen, Carlsbad, CA), and viewed with a Nikon Eclipse C1 confocal microscope using 488 and 561 nm filters to estimate biofilm thickness. An S3500N SEM (Hitachi, Japan) was also used to image biofilms and to verify uniform attachment over the entire electrode.
